# Aerobic Oil-Phase Cyclic Magnetic Adsorption to Synthesize 1D Fe_2_O_3_@TiO_2_ Nanotube Composites for Enhanced Visible-Light Photocatalytic Degradation

**DOI:** 10.3390/nano10071345

**Published:** 2020-07-09

**Authors:** Qingqing Tao, Xin Huang, Jingtao Bi, Rongli Wei, Chuang Xie, Yongzhu Zhou, Lu Yu, Hongxun Hao, Jingkang Wang

**Affiliations:** 1 National Engineering Research Center of Industry Crystallization Technology, School of Chemical Engineering and Technology, Tianjin University, Tianjin 300072, China; qqtao@tju.edu.cn (Q.T.); x_huang@tju.edu.cn (X.H.); jingtaob@gmail.com (J.B.); weirongli@tju.edu.cn (R.W.); acxie@tju.edu.cn (C.X.); jkwang@tju.edu.cn (J.W.); 2 Co-Innovation Center of Chemical Science and Engineering, Tianjin 300072, China; 3 Department of Chemistry, School of Science, Tianjin Chengjian University, Tianjin 300384, China; yzzhou@tcu.edu.cn

**Keywords:** 1D nanostructures, TiO_2_ nanotubes, iron oxides, visible-light photocatalysis

## Abstract

In this work, Fe_2_O_3_@TiO_2_ nanostructures with staggered band alignment were newly designed by an aerobic oil-phase cyclic magnetic adsorption method. XRD and TEM analyses were performed to verify the uniform deposition of Fe_2_O_3_ nanoparticles on the nanotube inner walls of TiO_2_. The steady-state degradation experiments exhibited that 1FeTi possessed the most superior performance, which might be ascribable to the satisfying dark adsorption capacity, efficient photocatalytic activity, ease of magnetic separation, and economic efficiency. These results indicated that the deposition of Fe_2_O_3_ into TiO_2_ nanotubes significantly enhanced the activity of Fe_2_O_3_, which was mainly ascribed to the Fe_2_O_3_-induced formation of staggered iron oxides@TiO_2_ band alignment and thus efficient separation of h^+^ and e^−^. Furthermore, the PL intensity and lifetime of the decay curve were considered as key criterions for the activity’s evaluation. Finally, the leaching tests and regeneration experiments were also performed, which illustrated the inhibited photodissolution compared with TiO_2_/Fe_3_O_4_ and stable cycling ability, enabling 1FeTi to be a promising magnetic material for photocatalytic water remediation.

## 1. Introduction

In recent years, water pollution has become an international environmental problem that constricts the development of human health, economy and sustainability [1,2]. To solve this problem, various efficient technologies have been developed and employed to purify wastewater, such as photocatalysis, adsorption, chemical precipitation, and membrane filtration [3,4,5,6]. Among them, photocatalysis has been regarded as a promising approach due to its convenient operation, effective remediation and low environmental impacts [7,8]. Among the well-studied photocatalysts, nano iron oxides including α-Fe_2_O_3_, γ-Fe_2_O_3_ and Fe_3_O_4_ have received extensive investigations and increasing attentions in the field of photocatalytic decontamination, benefiting from low cost, nontoxicity, large surface area, and especially strong absorption in the visible light region [9,10]. Moreover, the favorable magnetism of iron oxides makes the recovery of catalysts more convenient and economical via applying an external magnetic field for cyclic utilization after wastewater treatment [11].

However, the major drawbacks of iron oxides including low charge carriers’ mobility and rapid photogenerated electron–hole recombination rate restrict its practical applications in photocatalysis [12]. Consequently, various strategies were developed to enhance its photocatalytic properties, such as controlling diverse morphologies (e.g., nanoparticles, nanocubes and nanodiscs) [13], doping metal or non-metal ions such as Ca and I [14,15], and coupling with other semiconductors to generate satisfactory photocatalytic heterostructures. Among them, the coupling strategy with other semiconductors has been receiving extensive attentions since these heterostructures can efficiently suppress recombination rates and promote transportation rates of photo-generated charge carriers. For instance, the heterogeneous photocatalysts including α-Fe_2_O_3_@AgCO_3_ [16], γ-Fe_2_O_3_@Mn_3_O_4_ [17] and Fe_3_O_4_@TiO_2_ [18] all displayed significantly increased photocatalytic performance compared with pure iron oxides.

Among the various semiconductors combined with iron oxides, TiO_2_ has been extensively investigated for their stable, nontoxic and economical properties [19,20]. Particularly, abundant attentions have been given to 1D TiO_2_ nanotubes (abbreviated as TNT in this article) due to their unique 1D nanotube architectures accompanied with superior charge transport property and large internal surface to disperse doped iron oxides nanoparticles [21,22,23]. Especially, the constructed heterostructures of TiO_2_ and iron oxides could further enhance the separation of charge carriers in this heterostructure. Recently, the majority of the researches on Fe_x_O_y_@TNT nanocomposites focused on Fe_3_O_4_@TNT composites [24,25]. Unfortunately, the narrow bandgap of Fe_3_O_4_ brought serious photodissolution of Fe_3_O_4_, which would deactivate the composites and induce the decrease of photocatalytic performance [25]. As a result, iron oxide species with larger bandgaps such as Fe_2_O_3_ (bandgap ~2.3 eV) rather than Fe_3_O_4_ with narrow bandgap (0.1 eV), are more beneficial for the separation of photo-induced charge carriers in the heterojunctions [26,27]. On the other hand, Fe_2_O_3_ possesses higher chemical stability than Fe_3_O_4_ at room temperature, which can be oxidized in the presence of oxygen. From this point of view, it is reasonable to believe that introducing Fe_2_O_3_ nanoparticles into TiO_2_ nanotubes would significantly enhance the separation of charge carriers in Fe_2_O_3_@TiO_2_ heterojunctions. Furthermore, the excellent magnetic property of Fe_2_O_3_ would make them easier to be recycled and regenerated in the photocatalytic decontamination research, which is considered as a sustainable material for future applications [11]. Although Fe_2_O_3_@TNT composites have bright prospects and potential applications in photocatalytic water remediation, efficient methods to synthesize TiO_2_ nanotubes with uniform Fe_2_O_3_ nanoparticles deposition still need to be developed since the agglomeration of Fe_2_O_3_ nanoparticles would act as the recombination center and accelerate the destruction of excitons. The extensively used manner to prepare this heterostructure is the dipping method, which is limited by the low-dispersion loading and severe agglomeration under high deposition [28,29]. In our previous work, an anaerobic oil-phase cyclic magnetic adsorption (OCMA) method was developed to uniformly deposit Fe_3_O_4_ nanoparticles into TNT, which would provide inspiration to inhibit the agglomeration of Fe_2_O_3_ nanoparticles in this work. Especially, although the carbon species was introduced into the designed Fe_3_O_4_@TiO_2_ composites to inhibit the migration of e^-^ towards Fe_3_O_4_, 13.5% (in 3 h) of Fe_3_O_4_ in the prepared Fe_3_O_4_@C@TiO_2_ composites were still photo-dissolved, which might induce poor recycling performance [27]. As a result, the wide-bandgap Fe_2_O_3_ nanoparticles, instead of narrow-bandgap Fe_3_O_4_, were designed for fabricating Fe_2_O_3_@TiO_2_ composites with enhanced photocatalytic performance and inhibited photo dissolution. Especially, the designed Fe_2_O_3_@TiO_2_ heterostructures avoid the incorporation of carbon materials, which might have unassessed toxicity to the environment. Nevertheless, there was little work on the synthesis method and photocatalytic investigation of TiO_2_ nanotubes with uniform deposition of Fe_2_O_3_ nanoparticles. 

In this work, uniform 1D magnetic Fe_2_O_3_@TNT composites with excellent dispersive Fe_2_O_3_ deposition were newly synthesized through a new aerobic OCMA method. This new synthesizing method is more convenient and economical compared with other reported methods. Moreover, the photocatalytic performance of these nanocomposites containing different amounts of Fe_2_O_3_ under visible light irradiation were evaluated and discussed. The results indicated that there was an optimum amount of Fe_2_O_3_ deposition, which could be well explained by the physicochemical properties including Fe_2_O_3_ dispersion, PL intensity and charge carriers’ lifetime. Finally, the photo dissolution of the obtained composites was further examined by the leaching experiments and the results indicated superior repression of photo dissolution and stable regeneration of Fe_2_O_3_@TiO_2_ compared with Fe_3_O_4_@TiO_2_ and Fe_3_O_4_@C@TiO_2_.

## 2. Materials and Methods

### 2.1. Materials and Reagents

Titanium foil (10 × 25 mm^2^, 0.1 mm thickness, ≥99.5%) was purchased from Beijing Qianshuo Non-ferrous Metal Co., Ltd. (Beijing, China). FeCl_3_·6H_2_O (≥99 wt%), FeSO_4_·7H_2_O (≥99 wt%) and NH_4_F (≥99.5 wt%) were supplied by Aladdin Co., Ltd. (Tianjin, China). *n*-hexane (C_6_H_14_), ethanol (C_2_H_5_OH), ethylene glycol ((CH_2_OH)_2_), oleic acid (C_18_H_34_O_2_), Rhodamine B (RhB), and ammonia water (NH_3_·H_2_O, 25 wt%) were provided by Guangfu Co., Ltd. (Tianjin, China). All reagents were analytical grade and used as received without further purification.

### 2.2. Synthesis of TiO_2_ Nanotubes

Electrochemical anodization was applied to synthesize self-organized porous TNT [30]. Prior to anodization, the Ti foils were ultrasonically treated in *n*-hexane, followed with dipping in ethanol and deionized water respectively, and then dried in a nitrogen stream. Then, the Ti foils were anodized under constant potential (60 V) for 4 h in a two-electrode system where Ti served as the working electrode and platinum (Pt) foil served as the counter electrode. The electrolyte was an ethylene glycol solution containing NH_4_F (0.135 M) and H_2_O (2% in volume). After anodization, the as-anodized samples were ultrasonically cleaned in ethanol for 5 min to remove surface debris and then stored in hexane for further deposition.

### 2.3. Synthesis of Fe_3_O_4_@oleic Acid

Fe_3_O_4_@oleic acid nanoparticles were prepared by chemical co-precipitation method [31]. The procedure was as follows: FeCl_3_·6H_2_O (140 mmol) and FeSO_4_·7H_2_O (70 mmol) were dissolved into 100 mL deionized water and then the solution was heated to 80 °C in 20 min with vigorous stirring. Then, 3 mL oleic acid and 15 mL ammonia water were added rapidly into this solution. After stirring for 1 h, the black magnetic gel was cooled to 25 °C and separated by magnetic decantation. The precipitated particles were washed several times by ethanol to remove the excess oleic acid, followed by drying in vacuum oven at 60 °C for 12 h.

### 2.4. Synthesis of 1D Fe_2_O_3_@TiO_2_ Nanotube Composites

**Scheme 1:**
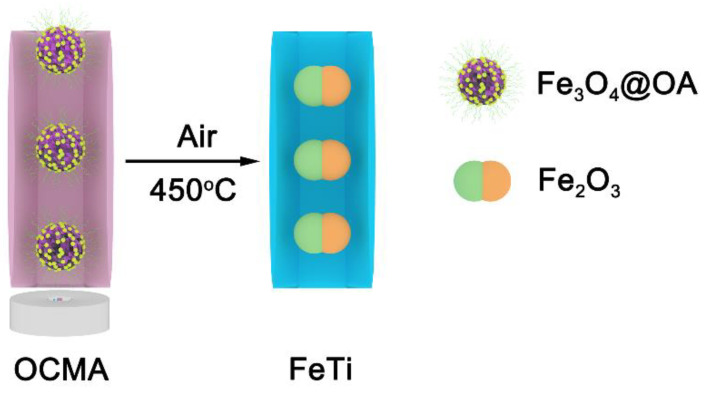
The preparation process of Fe_2_O_3_@TNT composites.

Fe_2_O_3_@TiO_2_ nanotube composites were first synthesized via an aerobic OCMA method [27]. Firstly, certain amounts of Fe_3_O_4_@oleic acid were ultrasonically dispersed in hexane and this suspension was filtered to remove the undispersed particles thoroughly and prepare a homogeneous solution of 1 g/L Fe_3_O_4_@oleic acid (The preparation of 1 g/L Fe_3_O_4_@oleic acid solution: Firstly, certain amounts (marked as *M*_1_) of Fe_3_O_4_@oleic acid were ultrasonically dispersed in hexane. Then, this suspension was filtered to remove the undispersed particles thoroughly. The mass of these undispersed particles can be determined and marked as *M*_2_. Therefore, the real amounts of Fe_3_O_4_@oleic acid dissolved in hexane can be calculated by $M_{1}-M_{2}$. After adding hexane to a certain volume, the concentration of “Fe_3_O_4_@oleic acid” in the homogeneous solution can be confirmed). Then, different volumes of the homogeneous solution (0.5 mL, 1 mL, 2 mL) were added onto the anodized TNT arrays under the effect of an external magnetic field beneath the TNT arrays, as shown in Scheme 1. It should be noticed that magnetic force played a vital role in loading Fe_3_O_4_@oleic acid nanoparticles into TiO_2_ nanotubes and the volatilization of hexane also made a contribution. Finally, these 0.5, 1, and 2 mL suspension-loaded TNT arrays were annealed at 450 °C for 2 h in air atmosphere to obtain Fe_2_O_3_@TNT composites with high crystallinity, which were recorded as 0.5FeTi, 1FeTi and 2FeTi, respectively. In addition, Fe_3_O_4_@oleic acid was also annealed at the same conditions to prepare pure Fe_2_O_3_ nanoparticles for comparative experiments.

### 2.5. Photocatalytic Experiments

For assessments of the photocatalytic activity of the samples, Rhodamine B (RhB) solutions in concentration of 10 mg/L were first prepared. Then, 70 mg as-prepared catalysts (Fe_2_O_3_, 0.5FeTi, 1FeTi and 2FeTi) were suspended into 70 mL RhB solutions, respectively. After dark adsorption for 2 h and reaching equilibrium, the mixed solutions were illuminated for 6 h under visible light (λ ≥ 400 nm) using a 300 W Xenon lamp (light intensity of 90 mW·cm^−2^ at distance of 15 cm from the light source). During the photocatalytic experiments, 3 mL samples of the mixed solutions were taken out and centrifuged at a given time interval. Finally, the photocatalytic activity was analyzed by UV−vis absorption measurement of the characteristic peak of RhB at 554 nm. For each sample, all photocatalytic experiments were carried out for irradiation duration of 6 h at 20 °C and were repeated three times.

### 2.6. Characterizations

The obtained samples were characterized by an XRD diffractometer (Rigaku, D/max 2500, Cu Kα radiation, λ = 1.5418 Å, Tokyo, Japan) within the scanning angle range of 15°–60° at rate of 8° min^−1^. The morphology was studied by scanning electron microscopy (SEM, Nanosem 430, FEI, Eindhoven, The Netherlands) and high-resolution transmission electron microscopy (HRTEM, Tecnai G20&F20, FEI, Eindhoven, The Netherlands). UV-Vis measurements were performed on a Hitachi U4100 UV Spectrometer and Fluorescence spectra were measured with FLS980 Series of Fluorescence Spectrometers (excited at λ = 420 nm). X-ray photoelectron spectroscopy (XPS, ESCALAB 250XI, Thermo, MA, USA) with monochromatic Al Kα (1486.6 eV) X-ray source was employed to analyze elemental contents and the chemical states of these composites. All the binding energies were calibrated by contaminant carbon at 284.8 eV. BET surface areas, BJH pore volume and average pore size were determined by N_2_ adsorption–desorption isotherms, which was measured by SSA-7000 (BJ Builder, Beijing, China). FTIR spectra were recorded on Bruker Alpha spectrometer from 4000 to 400 cm^−1^ (Regensburg, Germany). The magnetic properties were measured by vibrating sample magnetometer (VSM, MPMS SQUID XL, Quantum Design, Santiago, MN, USA) in the field from −20,000 to 20,000 Oe. High-resolution mass spectra (HR-MS) were measured by a mass spectrometer (Bruker, solanX 70 FT-MS, Regensburg, Germany) in electrospray ionization (ESI) mode.

## 3. Results and Discussion

### 3.1. Structural and Elemental Characterizations

**Figure 1:**
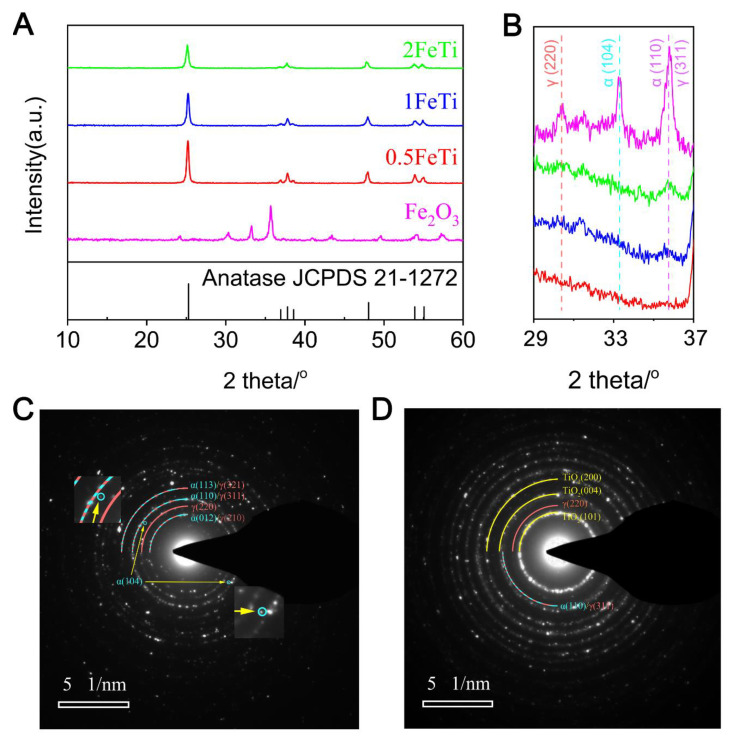
XRD full-range spectra (**A**) and narrow-range spectra (**B**) of Fe_2_O_3_ (magenta), 0.5FeTi (red), 1FeTi (blue) and 2FeTi (green); SAED images of Fe_2_O_3_ (**C**) and 2FeTi (**D**).

The crystal structures of these as-synthesized samples were determined by XRD analysis, as shown in Figure 1A. It can be found that the XRD patterns of as-prepared pure Fe_2_O_3_ contain diffraction peaks of both α-Fe_2_O_3_ at 33.2° (104) and γ-Fe_2_O_3_ at 30.2° (220), which indicates the coexistence of α-Fe_2_O_3_ and γ-Fe_2_O_3_ [32,33]. After the formation of a heterojunction, a series of new peaks, which can be well indexed to anatase TiO_2_ (JCPDS 21-1272), appear in the 1D Fe_2_O_3_@TNT composites. Since the peaks related to iron species were hardly to be observed due to the small amount of deposited Fe_2_O_3_ in Figure 1A, the narrow-range XRD spectra with higher resolution were further performed to examine the iron oxide species. As shown in Figure 1B, the characteristic peak intensity at 35.6° increases with the rising contents of Fe_2_O_3_ in 0.5FeTi, 1FeTi and 2FeTi, which further confirms the successful deposition of iron oxides. Furthermore, 2FeTi and Fe_2_O_3_ samples were further selected and analyzed by selective area electron diffraction (SAED) to ensure the existence of Fe_2_O_3_ (Figure 1C,D). As shown in Figure 1C, the SAED pattern of Fe_2_O_3_ with characteristic rings at 3.023 Å, 2.736 Å and 2.565 Å (from inside to outside) is in good agreement with the *d*-spacing of γ-Fe_2_O_3_ (*d*_220_ = 2.953 Å), α-Fe_2_O_3_ (*d*_104_ = 2.700 Å) and γ-Fe_2_O_3_ (*d*_311_ = 2.518 Å) [34]. For the SAED pattern of 2FeTi composite in Figure 1D, the diffractive rings of Fe_2_O_3_ are weak but still in agreement with corresponding crystal planes of Fe_2_O_3_, which might be due to the low and uniform dispersion of Fe_2_O_3_. Especially, it should be mentioned that the SAED patterns of 1FeTi and 0.5FeTi are also examined while the weak signals of Fe_2_O_3_ hinder their further characterization by this method. Nevertheless, the coexistence of α-Fe_2_O_3_ and γ-Fe_2_O_3_ in these composites is reasonable considering that all the samples are annealed at the same condition.

**Figure 2:**
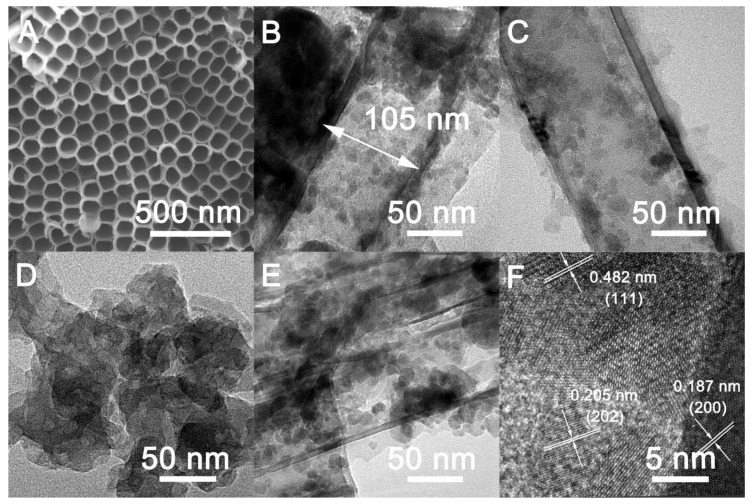
SEM and HRTEM morphologies for the samples. (**A**) SEM image of anodized TNT; (**B**) HRTEM image of 0.5FeTi; (**C**) HRTEM image of 1FeTi; (**D**) HRTEM image of Fe_2_O_3_; (**E**) HRTEM image of 2FeTi; (**F**) HRTEM image of 1FeTi.

To further confirm the inner structures of the prepared samples, SEM, TEM and HRTEM analyses were employed and the results are shown in Figure 2. SEM image of anodized TiO_2_ nanotubes in Figure 2A reveals that the nanotubes are highly ordered with a uniformed outer diameter distribution of ~108 nm. After doping with Fe_2_O_3_, the well dispersed black iron oxides nanoparticles on the inner walls of TNT can be clearly observed in the HRTEM image of 0.5FeTi, as presented in Figure 2B. The nanotubes with diameter of 105 nm also meet well with the SEM image in Figure 2A. With the increasing deposition, it is observed that the number of black Fe_2_O_3_ nanoparticles on the inner walls increases, as shown in Figure 2B,C,E. Furthermore, the Fe_2_O_3_ nanoparticles in 1FeTi (Figure 2C) and 2FeTi (Figure 2E) have similar inner structures with 0.5FeTi (Figure 2B) and are also well dispersed on TNT inner walls, indicating the successful deposition of Fe_2_O_3_ into TNT. Then, the crystalline forms of the synthesized composites were further examined by the HRTEM image of 1FeTi in Figure 2F. The lattice fringes with interplanar spacings of 0.187 nm, 0.205 nm and 0.482 nm are well ascribed to the (200) plane of TiO_2_, (202) plane of α-Fe_2_O_3_ and (111) plane of γ-Fe_2_O_3_, respectively, which is consistent with the observations from XRD. Finally, pure Fe_2_O_3_ nanoparticles were also examined by TEM (Figure 2D) and the aggregation of Fe_2_O_3_ particles is quite obvious, indicating the uniform dispersion could be easily achieved after doping into TiO_2_ nanotubes.

**Figure 3:**
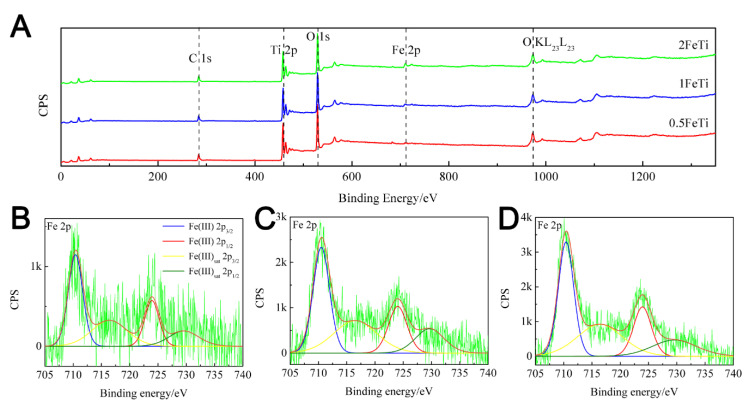
XPS spectra of the as-synthesized composites. (**A**) Full survey of samples; (**B**) Fe 2p region of 0.5FeTi; (**C**) Fe 2p region of 1FeTi; (**D**) Fe 2p region of 2FeTi.

After the determination of crystalline forms and inner structures of the synthesized composites, XPS analysis was carried out to further characterize the doping contents and surface chemical states of the obtained products, and the results are displayed in Figure 3. Figure 3A is the full-range spectra of the synthesized Fe_2_O_3_@TNT nanocomposites and the presence of C, Ti, O, Fe elements could be ascertained according to the peaks of C 1s, Ti 2p, O 1s, and Fe 2p. Specifically, the characteristic peak of C 1s situated at ~284.8 eV is attributed to the contaminant carbon, which is used for the calibration of the XPS spectra [35]. The Ti 2p_1/2_ and 2p_3/2_ peaks are observed at 464.1 and 458.0 eV respectively, which is in agreement with the typical anatase TiO_2_ [36]. The O 1s spectrum centered at 529.0 eV is ascribed to the metal oxides including Fe_2_O_3_ and TiO_2_. The Fe 2p spectra at 710.2 and 724.1 eV are the characteristic peaks of Fe_2_O_3_ and they were selected to analyze the chemical states of the doped Fe_2_O_3_ [37], as shown in Figure 3B–D. The Fe 2p spectra exhibit two contributions of 2p_1/2_ and 2p_3/2_ located at 723.9 and 710.4 eV, which can be assigned to Fe(III) oxidation state [37]. The additional peaks at 729.6 and 716.4 eV are ascribable to the satellite peaks of Fe(III) oxidation state. Especially, the peak intensity of Fe 2p increases with the growth of the Fe_2_O_3_ content, which is well consistent with the XRD results in Figure 1.

In order to identify the elemental molar ratios of the samples, careful calculation based on Figure 3A was performed after subtracting the peak of contaminant carbon, and the results are listed in Table 1. The O/Ti ratios in 0.5FeTi, 1FeTi and 2FeTi are 2.02, 2.17 and 2.31 respectively, which are close to theoretical values (2). Especially, the O/Ti ratios increase with the rising depositions, which further confirms the successful loading of Fe_2_O_3_ on the inner walls of TNT. It should be mentioned that the calculated Fe content at low deposition (0.5FeTi) was smaller than the theoretical value, which might be due to the fairly slight and highly dispersed Fe_2_O_3_ nanoparticles on the surface of TiO_2_ nanotubes. Except for the XPS detection, FTIR analysis was further carried out to characterize the chemical groups of the synthesized composites, which further confirms the successful deposition of Fe_2_O_3_ (shown in Figure S1). 

**Figure 4:**
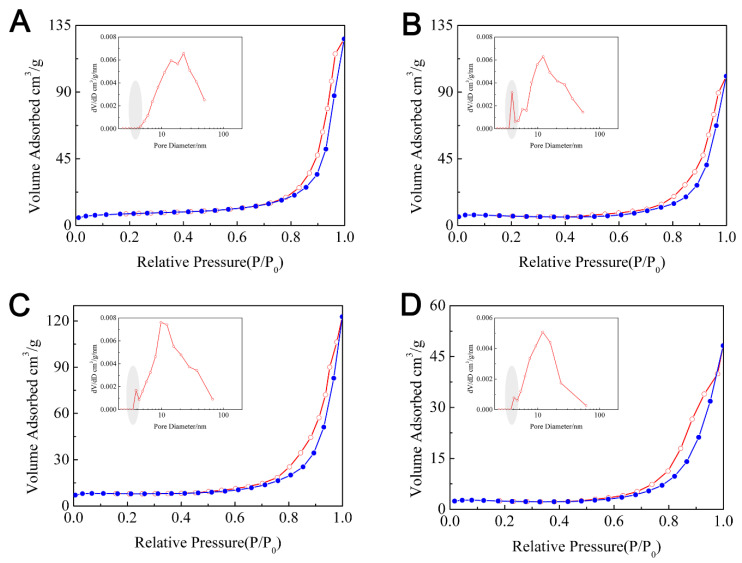
Nitrogen adsorption–desorption isotherms and the corresponding pore size distribution curves for 0.5FeTi (**A**), 1FeTi (**B**), 2FeTi (**C**), and Fe_2_O_3_ (**D**).

To further determine the inner structures of the synthesized nanocomposites, nitrogen adsorption/desorption isotherms analyses were utilized to examine the surface area and the pore information, and the results are presented in Figure 4 and Figure S2. The specific surface areas and pore sizes as well as volumes were calculated according to BET and BJH methods and the results are displayed in Table 2. Figure 4 shows that all the samples exhibit typical type IV isotherms with H_3_ hysteresis loop, suggesting the mesoporous structure of all the synthesized materials according to the IUPAC classification [38]. As shown in Table 2, the BET specific surface areas of these composites (0.5FeTi, 1FeTi, 2FeTi) increase significantly compared to bare Fe_2_O_3_. Considering the less BET-specific surface area of TNT (17.53 m^2^/g) than the composites, the enlargement of surface area is mainly ascribable to the uniformly doped Fe_2_O_3_ particles on TNT inner walls, which could not only enlarge the dispersibility of the iron oxides but also increase the contact area with the organic pollutants and thus enhanced photocatalytic performance. On the other hand, the BJH desorption branch pore sizes and volumes were selected to analyze the inner structures of the synthesized materials, as shown in Table 2. It can be observed that the pore sizes and volumes decrease with the increment of iron oxides, which also verifies the successful deposition of Fe_2_O_3_ in TiO_2_ nanotubes. The large pore size and pore volume of 0.5FeTi reveal that the Fe_2_O_3_ nanoparticles are highly dispersed on the inner walls of TiO_2_ nanotubes. With the increment of deposition, the pore sizes of 1FeTi and 2FeTi decrease sharply and fall into a range near pure Fe_2_O_3_, which is probably associated with the smaller inner structures between Fe_2_O_3_ nanoparticles, characterized at about 4 nm (as shown in the inset figure of Figure 4). Furthermore, it could be observed from the pore volume results of 1FeTi and 2FeTi that this value does not significantly decrease with the pore size, which further indicates more abundant pores in 1FeTi and 2FeTi and verifies the deduction mentioned above. Finally, it is observed that the peak area associated with Fe_2_O_3_ (marked by shadow ellipse in Figure 4) increases following this order: 1FeTi > 2FeTi > Fe_2_O_3_ > 0.5FeTi, which indicates the best deposition and dispersion of Fe_2_O_3_ nanoparticles in 1FeTi.

### 3.2. Photo-Chemical and Bandgap Characterizations

**Figure 5:**
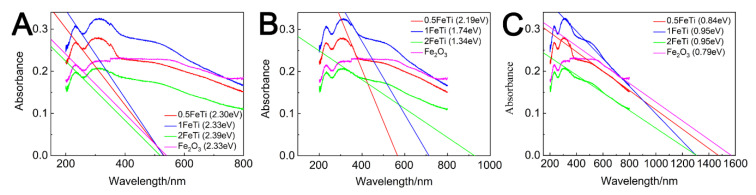
UV−vis absorption spectra and the bandgap correlations for 0.5FeTi, 1FeTi, 2FeTi, and Fe_2_O_3_ in the range of (**A**) 240–255 nm; (**B**) 354–380 nm; (**C**) 590–720 nm.

**Figure 6:**
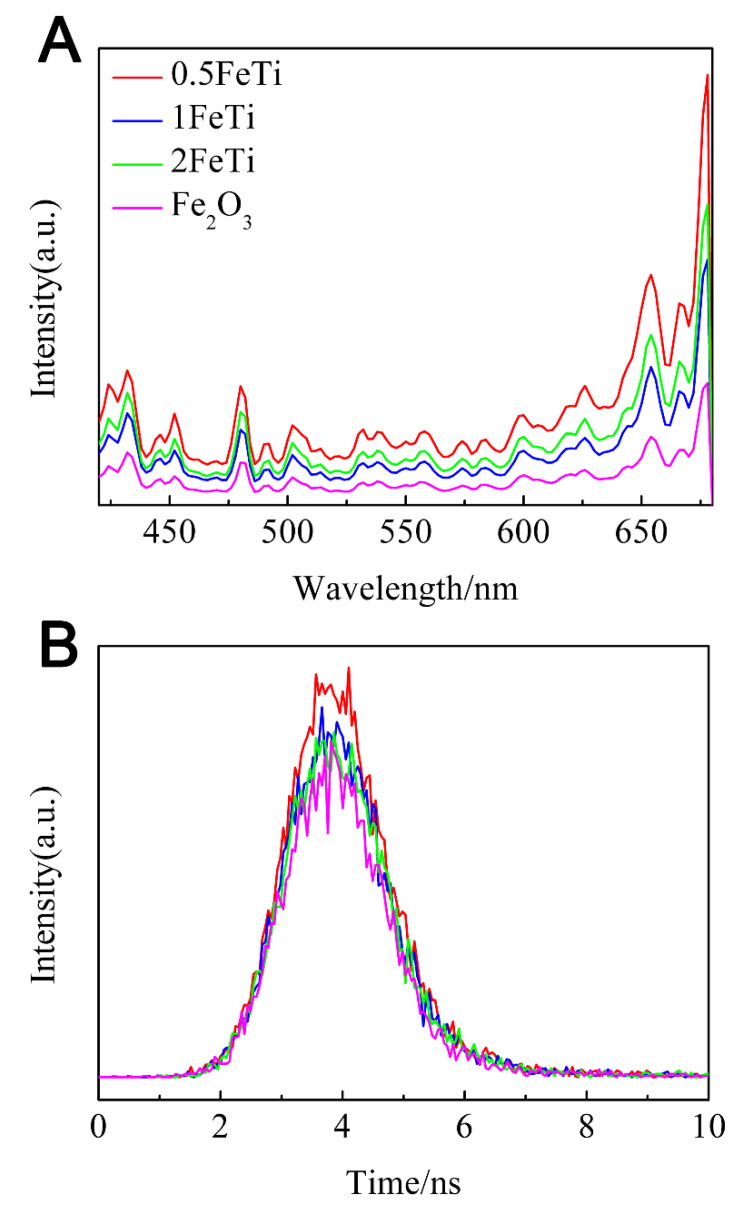
(**A**) PL spectra of 0.5FeTi, 1FeTi, 2FeTi, and Fe_2_O_3_; (**B**) Decay curves of 0.5FeTi, 1FeTi, 2FeTi, and Fe_2_O_3_ (excited at λ = 420 nm).

After the determination of the inner structures and chemical contents, the photo-chemical properties and bandgap information of the synthesized 1D nanocomposites were investigated through UV-Vis and PL spectra, as presented in Figure 5 and Figure 6.

Figure 5 shows the UV–vis absorption spectra of Fe_2_O_3_@TNT samples with variable Fe content and pure Fe_2_O_3_. The tangent lines at different wavelength range were used to determine the gap information of the synthesized materials. Fe_2_O_3_ possessed excellent absorption in both UV region and visible region. The tangent line of Fe_2_O_3_ in the wavelength from 240 nm to 255 nm correlated well with the bandgap of 2.33 eV, which is consistent with the reported value for Fe_2_O_3_ [26]. Besides the bandgap of Fe_2_O_3_ observed in Figure 5A, one more weak peak is observed in Figure 5C and the absorption bandgap energy can be extended to 0.79 eV, which is correlated with the midgap of Fe_2_O_3_. As for Fe_2_O_3_@TNT composites, the first peak that appeared in Figure 5A is mainly related to the doped iron oxides and it can be seen that the absorption bandgap energy improves slightly with increasing Fe content. Additionally, similar results are found in the third peak shown in Figure 5C. Furthermore, a new peak appeared in the range from 354 nm to 380 nm after the generation of Fe_2_O_3_@TNT heterojunction in Figure 5B, which is probably due to the migration of photogenerated charge carriers between TiO_2_ and Fe_2_O_3_. After the correlation, it was found that the derived bandgap energy in Figure 5B decreases significantly with increasing loading of iron oxides. 

**Scheme 2:**
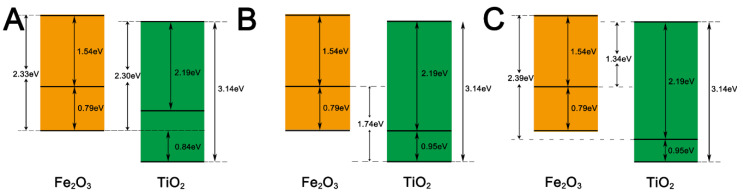
Staggered band alignments in as-prepared composites (**A**) 0.5FeTi; (**B**) 1FeTi; (**C**) 2FeTi.

Generally, single bandgap derived by UV-Vis spectrum cannot determine the band position, and further characterizations such as ultraviolet photoelectron spectroscopy (UPS) or Mott-Schottky measurement should be utilized to determine the position of valence band or conduction band [14,39]. Nevertheless, as the gaps derived in Figure 5B were regarded as the results of electron migration between Fe_2_O_3_ and TiO_2_, the relative positions of their individual gaps could be obtained. Thus, based on the extrapolation of UV–vis spectra in Figure 5, the band structures of these four catalysts were derived and are schematically shown in Scheme 2. It is interesting to note that the band alignment of these heterostructures are staggered form, which is not in accordance with the native included alignment of Fe_x_O_y_@TiO_2_ [40]. Such band alignments might be attributed to the coexistence α-Fe_2_O_3_ and γ-Fe_2_O_3_, resulting in the formation of staggered band alignment of α-Fe_2_O_3_/γ-Fe_2_O_3_ heterojunction. Finally, it should be mentioned that the staggered band form between Fe_2_O_3_ and TiO_2_ would significantly hinder the recombination of charge carriers and thus enhance the photocatalytic performance [41].

Figure 6 shows the PL spectra and decay curves of the synthesized materials. PL spectra were traditionally applied to detect the photo-chemical properties of the synthesized materials and it was considered that higher PL intensity indicated more electron-hole pairs generated in the heterostructures containing iron oxides [42]. Figure 6A clearly shows that the PL spectra of Fe_2_O_3_ nanoparticles is lower than that of three Fe_2_O_3_@TNT composites, implying that combining Fe_2_O_3_ with TiO_2_ nanotubes could effectively promote the generation of charge carriers. Except for the fluorescence intensity, the luminescence efficiency should also be considered by analyzing fluorescence lifetime. It can be observed in Figure 6B that the intensity of the decay curves fast diminishes after reaching the top of peak instead of a slow tailing trend, which might be due to the high charge carriers recombination rate of Fe_2_O_3_. Then, the fluorescence lifetimes of 0.5FeTi, 1FeTi, 2FeTi, and Fe_2_O_3_ were determined from the decay curves and fitted by a single exponential term. According to the corresponding equation (as shown in Figure S3), the lifetimes of 2FeTi, 1FeTi, 0.5FeTi, and Fe_2_O_3_ were calculated to be 0.6844 ns, 0.6759 ns, 0.6475 ns, and 0.6306 ns, respectively, indicating that the lifetimes also increase with the increment of Fe_2_O_3_ deposition. Additionally, these results also reveal the superb migration and effective separation of electrons and holes between Fe_2_O_3_ and TiO_2_, which is also shown in Scheme 2. Under the consideration of both charge carriers’ generation and their lifetimes, 2FeTi has the highest fluorescence intensity and fluorescence lifetime, which indicates that 2FeTi might have the best photocatalytic activity, followed by 1FeTi or 0.5FeTi and lastly bare Fe_2_O_3_.

### 3.3. Magnetic Characterizations

**Figure 7:**
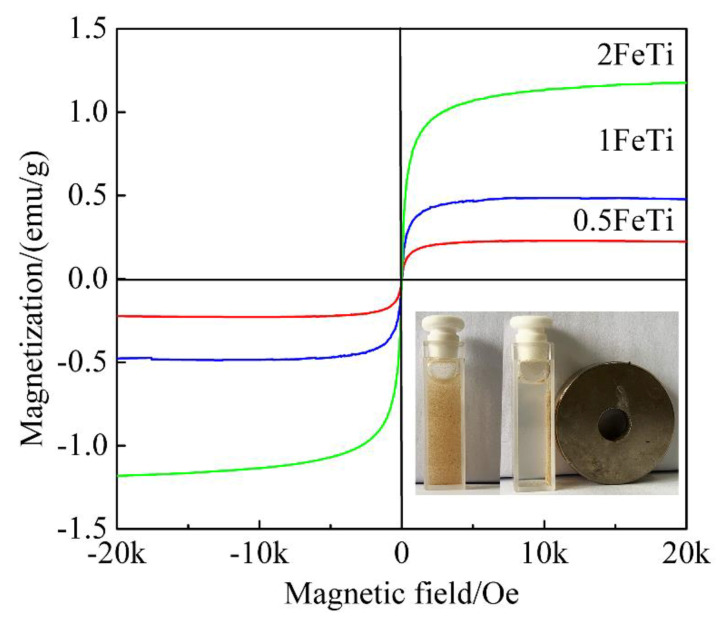
Magnetization curves of 0.5FeTi, 1FeTi and 2FeTi. (The inset is the magnetic separation process of 1FeTi in solution using a magnet).

Finally, the magnetic property of as-obtained products was evaluated by using a vibrating sample magnetometer, which is of great importance for practical applications. Figure 7 exhibits the magnetization curves measured at 300 K, and the insert graph shows the magnetic separation result of 1FeTi by using a magnet. The suspension containing well-dispersed particles of 1FeTi turned into clear solution without residues left in only several seconds via a magnet, which implies that the synthesized 1D Fe_2_O_3_@TNT composites possess good magnetic separation ability. In addition, the magnetization curves passing through the origin with the magnetic saturation (*Ms*) values of 0.23, 0.48 and 1.18 emu/g for 0.5FeTi, 1FeTi and 2FeTi indicated that they all possessed superparamagnetic behaviors. Accordingly, the molar contents of Fe_2_O_3_@TNT composites were estimated to be 1.06%, 2.23% and 5.54% for 0.5FeTi, 1FeTi and 2FeTi respectively, which is calculated by comparing the *Ms* values of the synthesized composites and pure Fe_2_O_3_ (the *Ms* value was 27.48 emu/g, shown in Figure S3). The results derived from *Ms* values are a little higher than the contents derived from the XPS spectra, which is ascribed to more excellent magnetism originating from the mini “magnet” formed along the nanotubes after the magnetic Fe_2_O_3_ nanoparticles are confined in the nanotubes. On the other hand, XPS technique is a surface-sensitive technique that excites electrons from the top surface of 1–12 nm thick, which might also be a reason for lower contents.

### 3.4. Photocatalytic Activity

**Figure 8:**
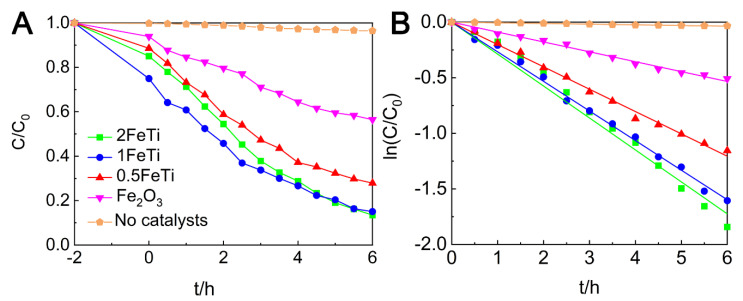
Photodegradation of RhB by Fe_2_O_3_ and Fe_2_O_3_@TNT composites under visible light irradiation: (**A**) Degradation rate curves; (**B**) Pseudo-first correlation of kinetic constants.

The photocatalytic activities of as-synthesized composites under visible light were assessed by the degradation experiments of RhB (10 mg/L) using Xe light irradiation (300 W, λ ≥ 400 nm). The variation of the concentration with the time of irradiation is plotted in Figure 8. During dark equilibrium period, these Fe_2_O_3_@TNT composites possess more efficient adsorption capacity for RhB compared with pure Fe_2_O_3_ and TNT (Figure S5), which is mainly ascribed to the homogeneous dispersity of Fe_2_O_3_ on TNT, as illustrated in Figure 2 and Figure 4. Especially, it is observed that 1FeTi shows the optimal adsorption property, which might have resulted from the most dispersive Fe_2_O_3_ inner structures in 1FeTi (Figure 4). After the visible-light irradiation, Fe_2_O_3_@TNT composites show remarkably enhanced photocatalytic activity compared to pure Fe_2_O_3_, which is attributed to the efficient separation of charge carriers after doping onto TiO_2_ nanotubes. Briefly, Fe_2_O_3_ would act as the electron trappers in the composites which would enhance separation of excitons and thus the photocatalytic performance. In addition, the performances of 1FeTi and 2FeTi are almost the same and much better than 0.5FeTi, which might be due to the more abundant electron trappers accompanied with the moderate increment of Fe_2_O_3_ deposition. Hence, 1FeTi and 2FeTi are considered as the candidates with the most potential for organics degradation. However, 1FeTi consumes less iron oxides and thus was more cost-effective. Above all, 1FeTi is considered to be a better Fe_2_O_3_@TNT composite under the consideration of dark adsorption capacity, photocatalytic activity and economic efficiency. Finally, the rate constant *k_a_* is fitted according to the pseudo-first order reaction kinetics (Equation (1)), and the results are displayed in Table 3. The order of the rate constants is in good agreement with the curves in Figure 8B. Interestingly, it is observed that the trend of rate constant is coincident with the deduction of the PL results, which implies that fluorescence intensity and fluorescence lifetime could describe the photocatalytic property of Fe_2_O_3_ and its composites effectively. Finally, the comparison of this study with other similar Fe_2_O_3_-based materials reported in other literatures is given in Table 4. As seen in Table 4, the photodegradation activity of 1FeTi is significantly improved, which further demonstrates that 1FeTi could be a promising material for photocatalytic wastewater remediation.

(1) $$\ln C_{0}/C=kt\;\;\;$$

where *C*_0_ and *C* are the original concentration of RhB and the corresponding concentration at the reaction time (*t*), respectively, and *k* is the pseudo-first degradation rate constant.

### 3.5. Intermediate Products and Degradation Pathway of RhB

**Scheme 3:**
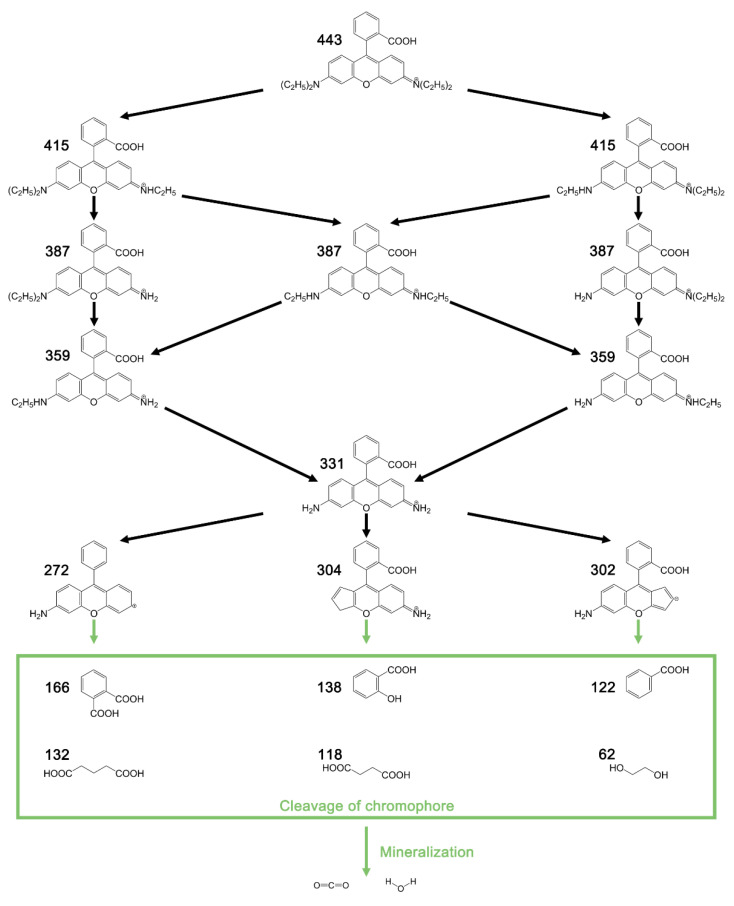
Plausible mineralization pathways for RhB by using 1FeTi.

Although it has been claimed that 84.96% of the RhB could be degraded by 1FeTi in 6 h in Section 3.4, the specific mineralization efficiency has not been determined. Consequently, the intermediate products during photocatalysis of RhB over 1FeTi for 0–6 h were identified by HR-MS. As shown in Figure S6, the HR-MS spectra of RhB solution (*t* = 0 h) shifted from *m/z* = 443 (characterized as RhB) to *m/z* = 415, 387, 359, 331, 304, 302, and 272 after photo illumination, which might be ascribable to the N-de-ethylating process under the effects of photogenerated radicals [49,50]. Then, the N-de-ethylated intermediates would react with functional radicals to produce opening-ring intermediates (e.g., phthalic acid), which could be further mineralized to CO_2_ and H_2_O [49,50]. Based on these deductions, the degradation pathway of RhB was proposed and is displayed in Scheme 3. Finally, it should be mentioned that the intensity of MS spectrum at *t* = 6 h is much weaker than that at *t* = 0 h, indicating the almost total mineralization of RhB in the wastewater.

### 3.6. Recyclable Performance

**Figure 9:**
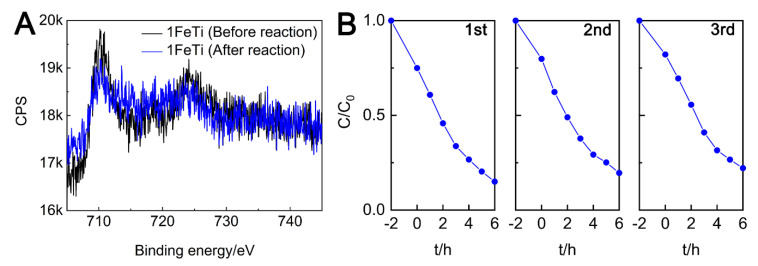
Recyclable performance of 1FeTi under visible light irradiation: (**A**) XPS Fe 2p spectra of 1FeTi before and after photodegradation; (**B**) Photodegradation performance of 1FeTi in the RhB solutions within three cycles.

Before the regeneration experiments, the leaching test should be probed to examine the loss of supported Fe_2_O_3_ particles and to ensure the maintenance of superior photocatalytic performance [27]. As a result, 1FeTi after photocatalysis was sampled and tested by XPS, and the results are displayed in Figure S7 and Figure 9A. It could be observed from the Fe 2p spectra in Figure 9A that peak shape and intensity is almost unchanged after photocatalysis, indicating the effective inhibition of photo dissolution of the doped Fe_2_O_3_ [25]. Furthermore, the dissolution amount was determined to be 13.4% in 6 h according to Table 1 and Figure S7, which is much less than 17.5% in 0.5 h for the Fe_3_O_4_@TiO_2_ and 13.5% in 3 h for Fe_3_O_4_@C@TiO_2_ heterostructures [25,27], verifying the hypothesis that the photo dissolution could be significantly hindered by incorporating wide-gap Fe_2_O_3_ instead of narrow-gap Fe_3_O_4_.

Considering the good stability of 1FeTi during photocatalysis, the regeneration experiments were performed. As shown in Figure 9B, the recyclable performance is almost stable in three cycles, and the slight reduction might be due to the moderate dissolution of Fe_2_O_3_ from the composite. Generally, 1FeTi exhibits superior repression for photo dissolution and stable regeneration, which could be considered as a promising photocatalytic medium for water remediation.

## 4. Conclusions

In this work, 1D Fe_2_O_3_@TiO_2_ nanotube composites were first successfully synthesized by an aerobic OCMA method. The structural and photo-chemical properties of the prepared Fe_2_O_3_ and Fe_2_O_3_@TNT composites were characterized via XRD, XPS, FTIR, HRTEM, BET, UV-Vis, and PL spectra. Based on the XRD results, both α-Fe_2_O_3_ and γ-Fe_2_O_3_ were found to exist in pure Fe_2_O_3_. The successful deposition of Fe_2_O_3_ was further confirmed by XPS and FTIR spectra. In addition, HRTEM was also applied to evaluate the dispersity of the Fe_2_O_3_ nanoparticles, and the results indicated that the nanoparticles were well dispersed on the TNT inner walls, resulting in efficient separation of charge carriers and homogeneous dispersion of Fe_2_O_3_ nanoparticles, which was further verified by the PL and BET analysis. According to the UV–vis spectra, the band structures of these catalysts were derived and it was found that the band alignments of these heterostructures were staggered, which might have resulted from the coexistence of α-Fe_2_O_3_ and γ-Fe_2_O_3_. Benefiting from the superior structural and photo-chemical properties, the composites exhibited much higher photocatalytic degradation efficiency towards RhB than pure Fe_2_O_3_ under visible-light irradiation. The photocatalytic results were in good agreement with PL results, which revealed the photoactivity tendency of the heterojunctions could be well predicted through fluorescence intensity and fluorescence lifetime. Furthermore, the introduction of Fe_2_O_3_ in Fe_2_O_3_@TNT nanostructures made them easy to be separated and recovered in a magnetic field, which further confirmed that the synthesized Fe_2_O_3_@TNT composites are efficient and cost-effective materials. Finally, the photo dissolution phenomenon of 1FeTi was examined and the low leaching amount as well as stable recycling performance illustrated the promising prospects of 1FeTi for water remediation and rational design of Fe_2_O_3_ into TiO_2_ instead of Fe_3_O_4_.

## Figures and Tables

**Table 1:** Molar content of carbon-subtracted peak area in Figure 3A.

Sample	Ti 2p/%	O 1s/%	Fe 2p/%
0.5FeTi	32.97	66.57	0.47
1FeTi	30.90	67.02	2.09
2FeTi	29.04	66.94	4.02

**Table 2:** BET surface area and BJH pore results of the as-prepared catalysts.

Sample	S_BET_/(m^2^·g^−^^1^)	Adsorption Branch		Desorption Branch	
		Pore Size/nm	Pore Volume/cm^3^·g^−^^1^	Pore Size/nm	Pore Volume/cm^3^·g^−^^1^
0.5FeTi	28.46	24.77	0.195	22.72	0.186
1FeTi	29.36	23.31	0.160	12.23	0.148
2FeTi	28.29	24.42	0.192	9.76	0.174
Fe_2_O_3_	10.96	13.77	0.079	11.99	0.069

**Table 3:** Photodegradation parameters of the synthesized catalysts.

Sample	0.5FeTi	1FeTi	2FeTi	Fe_2_O_3_
Degradation Efficiency (%)	72.11	84.96	86.53	43.55
*k*/h^−1^	0.2010	0.2663	0.2875	0.0890
*R^2^*	0.9985	0.9987	0.9936	0.9972

**Table 4:** Comparison of the photocatalytic performances of Fe_2_O_3_-based materials.

Materials	Light Source	Time (h)	Photodegradation Amount (%)	Ref.
α-Fe_2_O_3_	Visible light	3	38% MB	[43]
Fe_2_O_3_/SnO_2_	UV light	4	70% MB	[44]
α-Fe_2_O_3_/γ-Fe_2_O_3_	Visible light	12	90% RhB	[45]
γ-Fe_2_O_3_@TiO_2_	UV light	5	~18% 4-chlophenol	[46]
Fe_2_O_3_@WO_3_	Polychromatic light	3	18% RhB	[47]
Fe_2_O_3_	Visible light	6	~70% RhB	[48]
2FeTi	Visible light	6	86.53% RhB	This work

## Supplementary Materials

The following are available online at https://www.mdpi.com/2079-4991/10/7/1345/s1, Figure S1: N_2_ adsorption-desorption isotherms, Figure S2: Decay curve fitting procedures for the synthesized samples; Figure S3: Magnetization curve of Fe_2_O_3_.

## References

[1] Rasool K., Pandey R.P., Rasheed P.A., Buczek S., Gogotsi Y., Mahmoud K.A. (2019). Water treatment and environmental remediation applications of two- dimensional metal carbides (MXenes). Mater. Today.

[2] Shannon M.A., Bohn P.W., Elimelech M., Georgiadis J.G., Marĩas B.J., Mayes A.M. (2008). Science and technology for water purification in the coming decades. Nature.

[3] Ouyang J., Wang Y., Li T., Zhou L., Liu Z. (2018). Immobilization of carboxyl-modified multiwalled carbon nanotubes in chitosan-based composite membranes for U (VI) sorption. J. Radioanal. Nucl. Chem..

[4] Li G., Wang Y., Bi J., Huang X., Mao Y., Luo L. (2020). Partial Oxidation Strategy to Synthesize WS_2_/WO_3_ Heterostructure with Enhanced Adsorption Performance for Organic Dyes: Synthesis, Modelling, and Mechanism. Nanomaterials.

[5] Huang X., Yang J., Wang J., Bi J., Xie C. (2018). Design and synthesis of core e shell Fe_3_O_4_@PTMT composite magnetic microspheres for adsorption of heavy metals from high salinity wastewater. Chemosphere.

[6] Bi J., Huang X., Wang J., Tao Q., Lu H., Luo L., Li G., Hao H. (2020). Self-assembly of immobilized titanate films with different layers for heavy metal ions removal from wastewater: Synthesis, modeling and mechanism. Chem. Eng. J..

[7] Yang B., Bi W., Wan Y., Li X., Huang M., Yuan R., Ju H., Chu W., Wu X., He L. (2018). Surface etching induced ultrathin sandwich structure realizing enhanced photocatalytic activity. Sci. China Chem..

[8] Xu Q., Zhang L., Yu J., Wageh S., Al-Ghamdi A.A., Jaroniec M. (2018). Direct Z-scheme photocatalysts: Principles, synthesis, and applications. Mater. Today.

[9] Hitam C.N.C., Jalil A.A. (2020). A review on exploration of Fe_2_O_3_ photocatalyst towards degradation of dyes and organic contaminants. J. Environ. Manag..

[10] Wang J., Bai Z. (2017). Fe-based catalysts for heterogeneous catalytic ozonation of emerging contaminants in water and wastewater. Chem. Eng. J..

[11] Wu W., Jiang C., Roy V.A.L. (2015). Recent progress in magnetic iron oxide-semiconductor composite nanomaterials as promising photocatalysts. Nanoscale.

[12] Chen C., Duan F., Zhao S., Wang W., Yang F., Nuansing W., Zhang B., Qin Y., Knez M. (2019). Porous Fe_2_O_3_ nanotubes with α-γ phase junction for enhanced charge separation and photocatalytic property produced by molecular layer deposition. Appl. Catal. B Environ..

[13] Kusior A., Michalec K., Jelen P., Radecka M. (2019). Shaped Fe_2_O_3_ nanoparticles—Synthesis and enhanced photocatalytic degradation towards RhB. Appl. Surf. Sci..

[14] Demirci S., Yurddaskal M., Dikici T., Sarıoğlu C. (2018). Fabrication and characterization of novel iodine doped hollow and mesoporous hematite (Fe_2_O_3_) particles derived from sol-gel method and their photocatalytic performances. J. Hazard. Mater..

[15] Guo S., Wang H., Yang W., Fida H., You L., Zhou K. (2020). Scalable synthesis of Ca-doped α-Fe_2_O_3_ with abundant oxygen vacancies for enhanced degradation of organic pollutants through peroxymonosulfate activation. Appl. Catal. B Environ..

[16] Guo R., Qi X., Zhang X., Zhang H., Cheng X. (2019). Synthesis of Ag_2_CO_3_/α-Fe_2_O_3_ heterojunction and it high visible light driven photocatalytic activity for elimination of organic pollutants. Sep. Purif. Technol..

[17] Ma Q., Zhang X., Guo R., Zhang H., Cheng Q., Xie M., Cheng X. (2019). Persulfate activation by magnetic γ-Fe_2_O_3_/Mn_3_O_4_ nanocomposites for degradation of organic pollutants. Sep. Purif. Technol..

[18] Beketova D., Motola M., Sopha H., Michalicka J., Cicmancova V., Dvorak F., Hromadko L., Frumarova B., Stoica M., Macak J.M. (2020). One-Step Decoration of TiO_2_ Nanotubes with Fe_3_O_4_ Nanoparticles: Synthesis and Photocatalytic and Magnetic Properties. ACS Appl. Nano Mater..

[19] Peng Y., Li M., Zhang S., Nie G., Qi M., Pan B. (2015). Improved performance and prolonged lifetime of titania-based materials: Sequential use as adsorbent and photocatalyst. Sci. China Chem..

[20] Meng A., Zhang L., Cheng B., Yu J. (2019). Dual Cocatalysts in TiO_2_ Photocatalysis. Adv. Mater..

[21] Lee K., Mazare A., Schmuki P. (2014). One-dimensional titanium dioxide nanomaterials: Nanotubes. Chem. Rev..

[22] Wang M., Sun L., Lin Z., Cai J., Xie K., Lin C. (2013). P-n Heterojunction photoelectrodes composed of Cu2O-loaded TiO_2_ nanotube arrays with enhanced photoelectrochemical and photoelectrocatalytic activities. Energy Environ. Sci..

[23] Feng J.X., Xu H., Dong Y.T., Lu X.F., Tong Y.X., Li G.R. (2017). Efficient Hydrogen Evolution Electrocatalysis Using Cobalt Nanotubes Decorated with Titanium Dioxide Nanodots. Angew. Chem. Int. Ed..

[24] Challagulla S., Nagarjuna R., Ganesan R., Roy S. (2016). Acrylate-based Polymerizable Sol-Gel Synthesis of Magnetically Recoverable TiO_2_ Supported Fe_3_O_4_ for Cr(VI) Photoreduction in Aerobic Atmosphere. ACS Sustain. Chem. Eng..

[25] Li Z.J., Huang Z.W., Guo W.L., Wang L., Zheng L.R., Chai Z.F., Shi W.Q. (2017). Enhanced Photocatalytic Removal of Uranium(VI) from Aqueous Solution by Magnetic TiO_2_/Fe_3_O_4_ and Its Graphene Composite. Environ. Sci. Technol..

[26] Ong W.J., Tan L.L., Ng Y.H., Yong S.T., Chai S.P. (2016). Graphitic Carbon Nitride (g-C3N4)-Based Photocatalysts for Artificial Photosynthesis and Environmental Remediation: Are We a Step Closer to Achieving Sustainability?. Chem. Rev..

[27] Bi J., Huang X., Wang J., Wang T., Wu H., Yang J., Lu H., Hao H. (2019). Oil-phase cyclic magnetic adsorption to synthesize Fe_3_O_4_@C@TiO_2_-nanotube composites for simultaneous removal of Pb(II) and Rhodamine B. Chem. Eng. J..

[28] Chen H., Li T., Zhang L., Wang R., Jiang F., Chen J. (2015). Pb(II) adsorption on magnetic γ-Fe_2_O_3_/titanate nanotubes composite. J. Environ. Chem. Eng..

[29] Zhao L., Cao Q., Wang A., Duan J., Zhou W., Sang Y., Liu H. (2018). Iron oxide embedded titania nanowires—An active and stable electrocatalyst for oxygen evolution in acidic media. Nano Energy.

[30] Roy P., Berger S., Schmuki P. (2011). TiO_2_ nanotubes: Synthesis and applications. Angew. Chem. Int. Ed..

[31] Yang K., Peng H., Wen Y., Li N. (2010). Re-examination of characteristic FTIR spectrum of secondary layer in bilayer oleic acid-coated Fe_3_O_4_ nanoparticles. Appl. Surf. Sci..

[32] Jang J.T., Lee J., Seon J., Ju E., Kim M., Kim Y.I.L., Kim M.G., Takemura Y., Arbab A.S., Kang K.W. (2018). Giant Magnetic Heat Induction of Magnesium-Doped γ-Fe_2_O_3_ Superparamagnetic Nanoparticles for Completely Killing Tumors. Adv. Mater..

[33] Liu S., Zheng L., Yu P., Han S., Fang X. (2016). Novel Composites of α-Fe_2_O_3_ Tetrakaidecahedron and Graphene Oxide as an Effective Photoelectrode with Enhanced Photocurrent Performances. Adv. Funct. Mater..

[34] Wu H., Wu G., Wang L. (2015). Peculiar porous α-Fe_2_O_3_, γ-Fe_2_O_3_ and Fe_3_O_4_ nanospheres: Facile synthesis and electromagnetic properties. Powder Technol..

[35] Ling M., Blackman C.S. (2018). Gas-phase synthesis of hybrid nanostructured materials. Nanoscale.

[36] Shen G., Zhang R., Pan L., Hou F., Zhao Y., Shen Z., Mi W., Shi C., Wang Q., Zhang X. (2020). Regulating the Spin State of Fe III by Atomically Anchoring on Ultrathin Titanium Dioxide for Efficient Oxygen Evolution Electrocatalysis Angewandte. Angew. Chem. Int. Ed..

[37] Xie J., Jin R., Li A., Bi Y., Ruan Q., Deng Y., Zhang Y., Yao S., Sankar G., Ma D. (2018). Highly selective oxidation of methane to methanol at ambient conditions by titanium dioxide-supported iron species. Nat. Catal..

[38] Mou H., Song C., Zhou Y., Zhang B., Wang D. (2018). Design and synthesis of porous Ag/ZnO nanosheets assemblies as super photocatalysts for enhanced visible-light degradation of 4-nitrophenol and hydrogen evolution. Appl. Catal. B Environ..

[39] Guo T., Wang K., Zhang G., Wu X. (2019). A novel α-Fe_2_O_3_@g-C3N4 catalyst: Synthesis derived from Fe-based MOF and its superior photo-Fenton performance. Appl. Surf. Sci..

[40] Puthirath Balan A., Radhakrishnan S., Woellner C.F., Sinha S.K., Deng L., Reyes C.D.L., Rao B.M., Paulose M., Neupane R., Apte A. (2018). Exfoliation of a non-van der Waals material from iron ore hematite. Nat. Nanotechnol..

[41] Chen Y., Jiang D., Li L., Li Z., Li Q., Shi R. (2020). Enhanced photoelectrochemical activity of α-Fe_2_O_3_/TiO_2_ nanoheterojunction by controlling hydrodynamic conditions. Nanotechnology.

[42] Charanpahari A., Umare S.S., Gokhale S.P., Sudarsan V., Sreedhar B., Sasikala R. (2012). Enhanced photocatalytic activity of multi-doped TiO_2_ for the degradation of methyl orange. Appl. Catal. A Gen..

[43] Liang H., Liu K., Ni Y. (2015). Synthesis of mesoporous α-Fe_2_O_3_ via sol-gel methods using cellulose nano-crystals (CNC) as template and its photo-catalytic properties. Mater. Lett..

[44] Lei R., Ni H., Chen R., Zhang B., Zhan W., Li Y. (2017). Growth of Fe_2_O_3_/SnO_2_ nanobelt arrays on iron foil for efficient photocatalytic degradation of methylene blue. Chem. Phys. Lett..

[45] Wei Z., Wei X., Wang S., He D. (2014). Preparation and visible-light photocatalytic activity of α-Fe_2_O_3_/γ-Fe_2_O_3_ magnetic heterophase photocatalyst. Mater. Lett..

[46] Tyrpekl V., Vejpravová J.P., Roca A.G., Murafa N., Szatmary L., Nižňanský D. (2011). Magnetically separable photocatalytic composite γ-Fe_2_O_3_@TiO_2_ synthesized by heterogeneous precipitation. Appl. Surf. Sci..

[47] Costa G.S., Costa M.J.S., Oliveira H.G., Lima L.C.B., Luz G.E., Cavalcante L.S., Santos R.S. (2020). Effect of the applied potential condition on the photocatalytic properties of Fe_2_O_3_/WO_3_ heterojunction films. J. Inorg. Organomet. Polym. Mater..

[48] Park Y., Kim H., Lee G., Pawar R.C., Lee J., Lee C.S. (2016). Photocatalytic evaluation of self-assembled porous network structure of ferric oxide film fabricated by dry deposition process. Mater. Chem. Phys..

[49] He Z., Sun C., Yang S., Ding Y., He H., Wang Z. (2009). Photocatalytic degradation of rhodamine B by Bi2WO6 with electron accepting agent under microwave irradiation: Mechanism and pathway. J. Hazard. Mater..

[50] Natarajan T.S. (2013). Enhanced photocatalytic activity of bismuth-doped TiO_2_ nanotubes under direct sunlight irradiation for degradation of Rhodamine B dye. J. Nanoparticle Res..

