# Supplementary material for: Aerobic Oil-Phase Cyclic Magnetic Adsorption to Synthesize 1D Fe2O3@TiO2 Nanotube Composites for Enhanced Visible-Light Photocatalytic Degradation
Source: Nanomaterials (Basel). 2020 Jul 9;10(7):1345. doi: 10.3390/nano10071345 (PMC7408372; doi:10.3390/nano10071345)
Supplement: Supplementary file 1 [file nanomaterials-10-01345-s001.pdf]

nanomaterials

Supplementary Materials

Aerobic Oil-Phase Cyclic Magnetic Adsorption to Synthesize 1D Fe_2O_3@TiO_2 Nanotube Composites for Enhanced Visible-Light Photocatalytic Degradation

Qingqing Tao [1], Xin Huang [1,2], Jingtao Bi [1], Rongli Wei [1], Chuang Xie [1,2], Yongzhu Zhou [3], Lu Yu [3,*], Hongxun Hao [1,2,*] and Jingkang Wang [1,2]

1 National Engineering Research Center of Industry Crystallization Technology, School of Chemical Engineering and Technology, Tianjin University, Tianjin 300072, China; qqtao@tju.edu.cn (Q.T.); x_huang@tju.edu.cn (X.H.); jingtaob@gmail.com (J.B.); weirongli@tju.edu.cn (R.W.); acxie@tju.edu.cn (C.X.); jkwang@tju.edu.cn (J.W.)
2 Co-Innovation Center of Chemical Science and Engineering, Tianjin 300072, China
3 Department of Chemistry, School of Science, Tianjin Chengjian University, Tianjin 300384, China; yzzhou@tcu.edu.cn
* Correspondence: yulu@tcu.edu.cn (L.Y.); hongxunhao@tju.edu.cn (H.H.)

S1 FTIR spectra of the synthesized samples

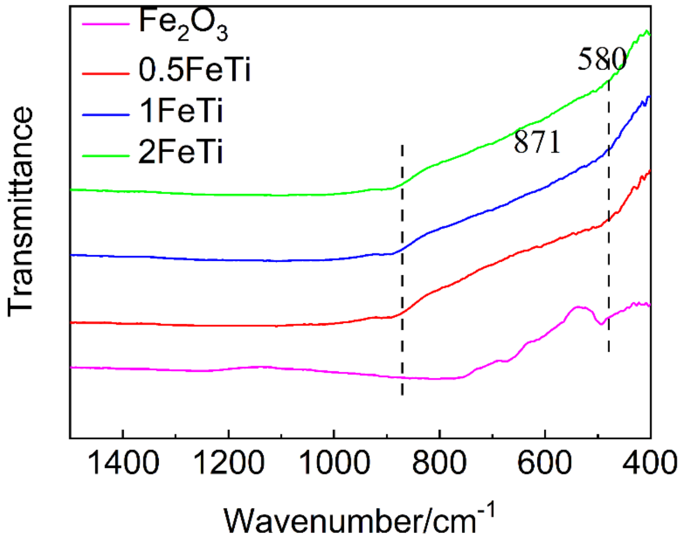

Figure S1. FTIR spectra of 0.5FeTi (red), 1FeTi (blue), 2FeTi (green) and Fe_2O_3 (magenta).

Note: The peaks at 871 cm^{-1} and 580 cm^{-1} were attributed to the stretching vibration of the Ti–O and the stretching vibration of Fe–O, respectively. Besides, a weak fluctuation appearing at 580 cm^{-1} is observed in 0.5FeTi, 1FeTi and 2FeTi, which also confirms the successful deposition of Fe_2O_3.

S2 N_2 adsorption-desorption isotherms

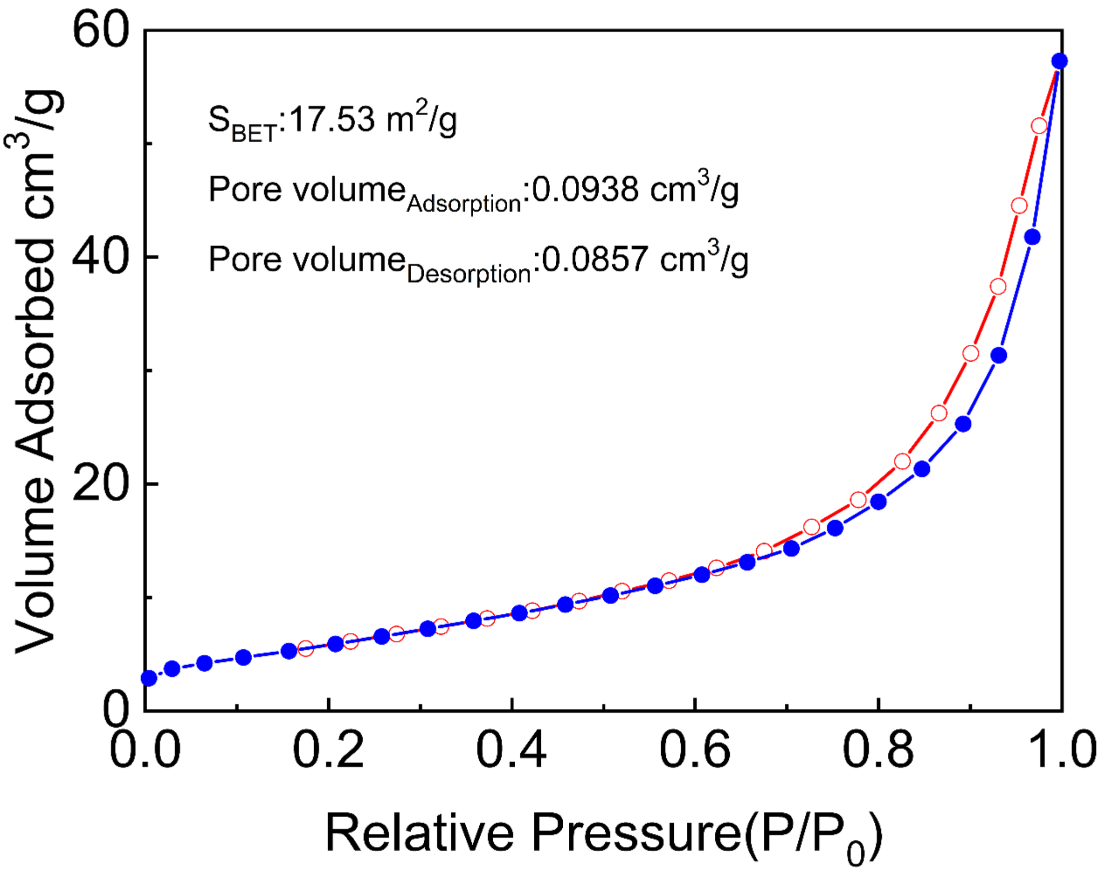

Figure S2. Nitrogen adsorption–desorption isotherms and the corresponding pore size distribution curves for TNT.

S3 Decay curve fitting procedures for the synthesized samples.

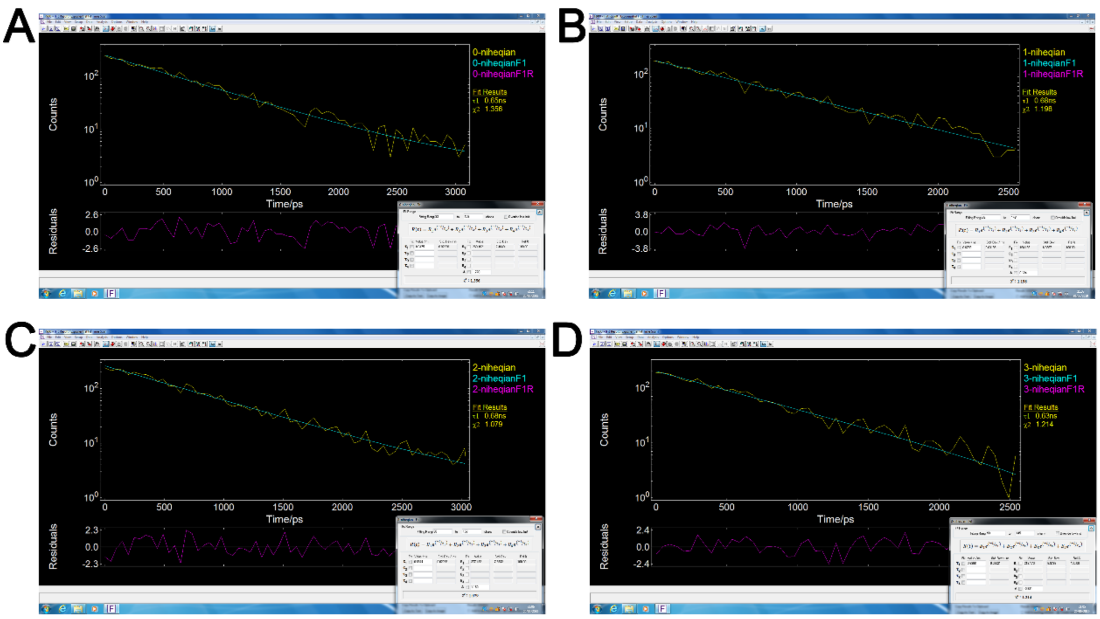

Figure S3. Decay curve fitting procedures for 0.5FeTi (**A**), 1FeTi (**B**), 2FeTi (**C**) and Fe_2O_3 (**D**).

S4 Magnetization curve of Fe_2O_3

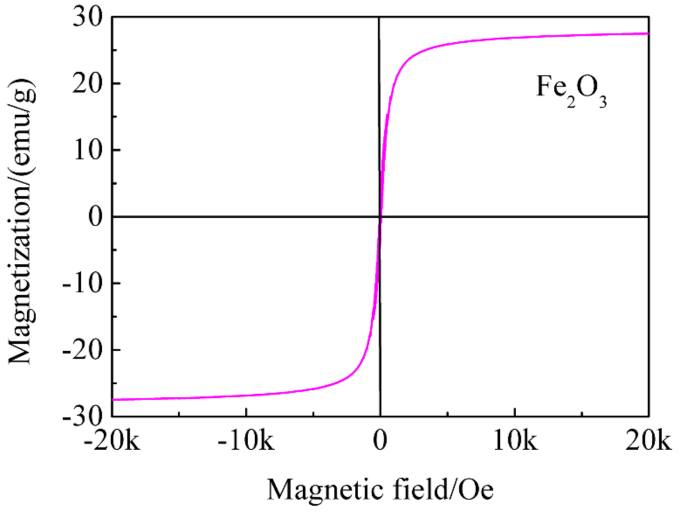

Figure S4. Magnetization curve of Fe_2O_3.

S5 Degradation curve of pure TiO_2 nanotubes

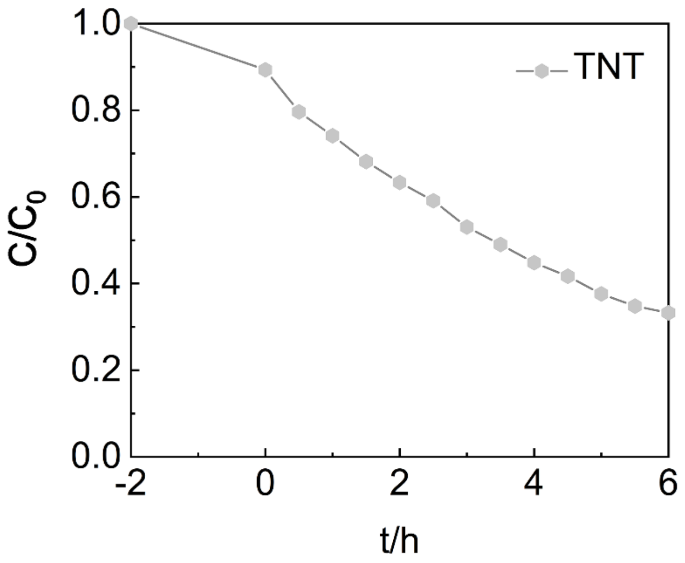

Figure S5. Degradation curve of pure TiO_2 nanotubes.

S6 HR-MS spectra

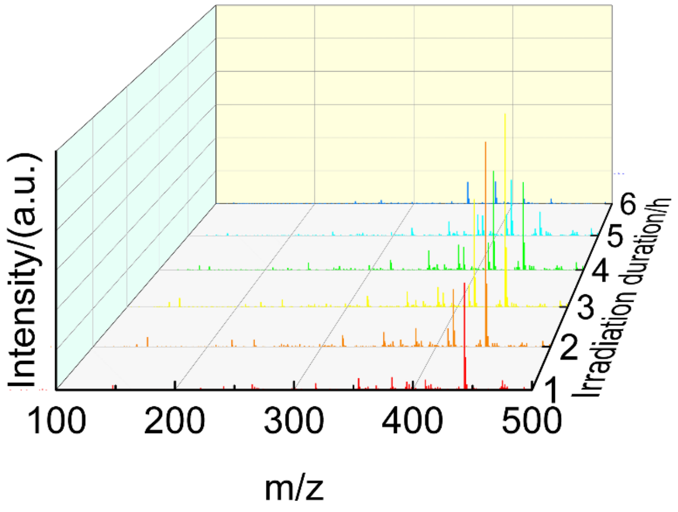

Figure S6. HR-MS spectra of samples during photocatalysis of RhB over 1FeTi for 0–6 h.

S7 XPS full-range spectrum of 1FeTi after photocatalysis

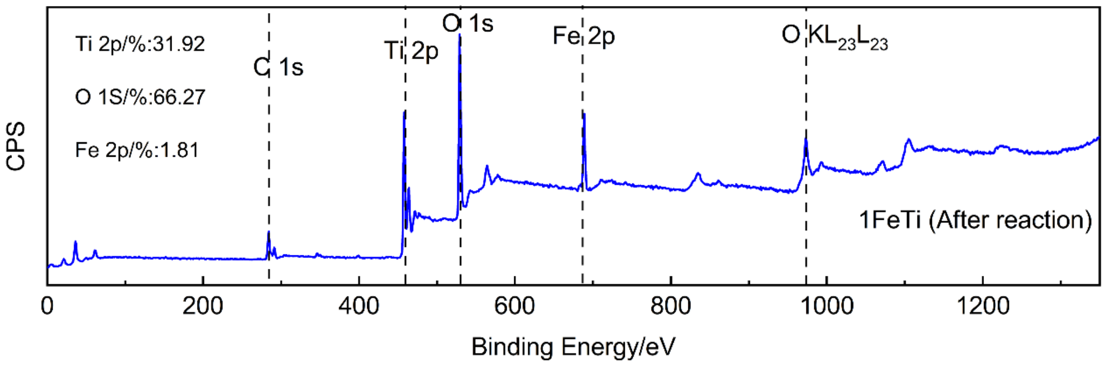

Figure S7. XPS full-range spectrum of 1FeTi after photocatalysis.

Note: the loss of iron can be calculated to be 1–1.81/2.09 = 13.4% according to Table 1 and Figure S7.
